# ddSeeker: a tool for processing Bio-Rad ddSEQ single cell RNA-seq data

**DOI:** 10.1186/s12864-018-5249-x

**Published:** 2018-12-24

**Authors:** Dario Romagnoli, Giulia Boccalini, Martina Bonechi, Chiara Biagioni, Paola Fassan, Roberto Bertorelli, Veronica De Sanctis, Angelo Di Leo, Ilenia Migliaccio, Luca Malorni, Matteo Benelli

**Affiliations:** 1 Bioinformatics Unit, Hospital of Prato, Prato, Italy; 2 Sandro Pitigliani Translational Research Unit, Hospital of Prato, Prato, Italy; 3 Sandro Pitigliani Medical Oncology Department, Hospital of Prato, Prato, Italy; 4 NGS Core Facility, Centre for Integrative Biology (CIBIO), University of Trento, Trento, Italy

**Keywords:** Single-cell transcriptomics, scRNA-seq, Bioinformatics

## Abstract

**Background:**

New single-cell isolation technologies are facilitating studies on the transcriptomics of individual cells. Bio-Rad ddSEQ is a droplet-based microfluidic system that, when coupled with downstream Illumina library preparation and sequencing, enables the monitoring of thousands of genes per cell. Sequenced reads show unique features that do not permit the use of freely available tools to perform single cell demultiplexing.

**Results:**

We present ddSeeker, a tool to perform initial processing and quality metrics of reads generated through Bio-Rad ddSEQ/Illumina experiments. Its application to the Illumina test dataset demonstrates that ddSeeker performs better than Illumina BaseSpace software, enabling a higher recovery of valid reads. We also show its utility in the analysis of an in-house dataset including two read sets characterized by low and high sequencing quality. ddSeeker and its source code are available at https://github.com/cgplab/ddSeeker.

**Conclusions:**

ddSeeker is a freely available tool to perform initial processing and quality metrics of reads generated through Bio-Rad ddSEQ/Illumina single cell transcriptomic experiments.

**Electronic supplementary material:**

The online version of this article (10.1186/s12864-018-5249-x) contains supplementary material, which is available to authorized users.

## Background

**Fig. 1:**
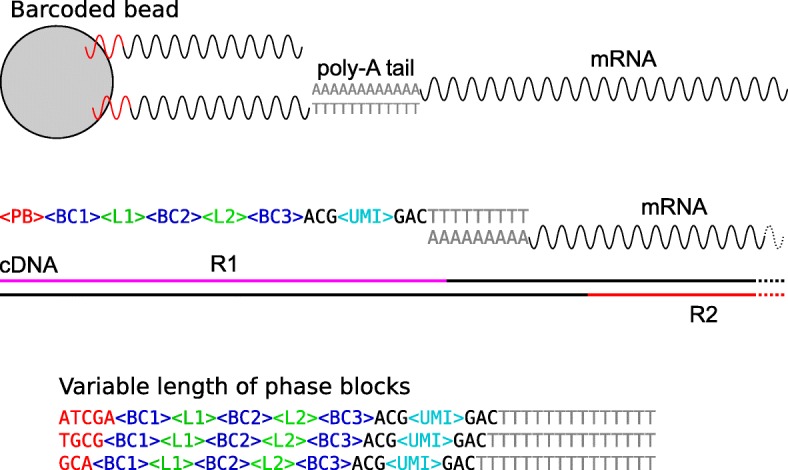
Schematic of Bio-Rad ddSEQ/Illumina reads’ structure (Top) In BioRad ddSEQ, barcoded beads capture mRNA molecules through hybridization with mRNA poly-A tails. Each single DNA strand is characterized by the following structure: a phase block (PB), three barcode blocks (BC1, BC2, BC3) interlinked by two different linkers (L1 and L2), and one UMI flanked by two trinucleotides (ACG and GAC). (Middle) Read 1 (R1) contains molecular tags while Read 2 (R2) contains the information of the mRNA sequence (R1 and R2 are not in scale). (Bottom) Separation of cDNA from the beads can occur at different nucleotides within the PB, thus making the position of the two linkers variable

Recent advances in single-cell transcriptome profiling (single cell RNA-seq, scRNA-seq), are improving our understanding of different biological processes, with impact in many areas of research, including the immune system, brain and mammal development and cancer [1, 2]. scRNA-seq techniques are contributing to refine our knowledge of cell types and states [3, 4] and have been successfully used to characterize intratumoral heterogeneity in different tumor types, including glioblastoma [5], melanoma [6] and breast cancer [7]. Clinical application of scRNA-seq has been also investigated by various groups. Most of these studies have focused on dissecting the interplay between tumor cell biology and cancer treatment, with the ultimate goal of identifying new treatment hypotheses [6, 8, 9]. A recent example includes the application of scRNA-seq to triple negative breast cancer patients in order to understand clonal evolution in response to chemotherapy [10].A variety of computational tools have been designed to address the specific challenges of scRNA-seq data. These involve new normalization methods for dealing with the small number of reads per gene and cell [11, 12], imputation strategies to model the sparsity of the data [13, 14], statistical methods to perform differential expression analysis [15, 16] and clustering techniques to capture cell population heterogeneity [17] and cell population dynamics [18]. Several platforms enabling single-cell transcriptome profiling are based on droplet-microfluidic technology, wherein cells are encapsulated into nanoliter droplets then lysed and mRNA is barcoded [19]. Available commercial systems include 10x Genomics Chromium [20] and Bio-Rad ddSEQ Single Cell Isolator [21]. In particular, ddSEQ is a new platform capable of isolating and barcoding about 300 cells per well. Libraries are then prepared by a specific Illumina kit (SureCell WTA 3’ [22]) and sequenced. Though in its infancy, the Bio-Rad ddSEQ platform has been successfully used to characterize the molecular heterogeneity of cystic precursor lesions (IPMNs) and its role towards progressive dysplastic changes [23]. ddSEQ data have been also exploited for validating new computational methods [16, 24].Popular tools for the processing of scRNA-seq data include Drop-seq tools [25], dropSeqPipe [26], dropEst [27], scPipe [28] and zUMI [29]. The first essential step in scRNA-seq is the identification of cell-specific barcodes. In Bio-Rad ddSEQ/Illumina cell barcoding, cells are identified by the combination of three barcodes of 6 nucleotides in length. However, compared to the other available platforms, the position of barcodes within Read 1 is not fixed due to the presence of phase blocks (Fig. 1). This unique feature renders the use of currently freely available tools for these data impossible; to date, the only available and feasible tool is a commercial software integrated in the suite of Illumina BaseSpace tools [30]. Here, we present ddSeeker, a new tool for the initial processing of data generated through Bio-Rad ddSEQ experiments that shows enhanced performance compared to the available commercial software. Our tool can be integrated in different scRNA-seq pipelines, allowing the users to take advantage of popular pipelines for the processing of scRNA-seq data. Additionally, ddSeeker provides a set of metrics which can assist the user to evaluate the quality of their own data.

## Implementation

Our method is implemented following the Illumina recommendations for the analysis of Read 1 (R1) structure. As reported in Fig. 1, we expect R1 to contain the molecular tags to identify both single cells (cell barcodes) and single transcript molecules (Unique Molecular Identifiers, UMI), while Read 2 (R2) contains the mRNA sequence. To identify reads with correct molecular tags (valid reads), our method implements the following steps:

1. the exact positions of the two linkers (L1 and L2) within the sequence of R1 are retrieved;
2. the distance between the starting positions of the two linkers is verified to be exactly 21 nt;
3. L1 is verified to start at least 7 nucleotides from the start of the sequence;
4. the sequences of the two trinucleotides flanking the UMI are verified to be ACG and GAC;
5. the sequences of the three barcode blocks (BC1, BC2, BC3) and the UMI are extracted based on their position relative to the linkers;
6. the sequences of the barcode blocks are compared with a predefined list of known barcode blocks, and retrieved only if a match is found.

In order to optimize the accuracy of our pipeline, we also considered insertions and deletions in the analysis of the sequences of linkers and barcodes (step 1 and 6) and at most one mismatch. Indeed, based on the analysis reported in Additional file 1: Figure S1 and Table S1, we observed that events with more than one mismatch, insertion or deletion in linkers represent a small fraction of total events (1.4% for L1 and 1.2% for L2). In step 4 only one mismatch and no indels are permitted due to the short length of sequences to be tested. For each valid read, the cell barcode is defined as the union of the three barcodes (BC1 + BC2 + BC3). Cell identifier and UMI are then associated to the corresponding R2. ddSeeker takes R1 and R2 fastq files as input and outputs an unmapped BAM file containing R2 and corresponding cell barcode and UMI molecular tags. ddSeeker uses as default a Drop-seq tools -like tag scheme (XC for cell barcode and XM for UMI), but different tag schemes can be chosen by the user. For non-valid reads, ddSeeker reports the error identifier in a custom defined tag XE (see Additional file 1 for the description of possible errors). Our tool is written in Python3 using Biopython [31] and pysam modules [32]. ddSeeker can be integrated with existing scRNA-seq pipelines, including Drop-seq tools, dropEst and scPipe. A detailed description of the algorithm is reported in Additional file 1. Analyses were made using R custom scripts [33] and plots generated by the ‘Tidyverse’ packages [34].

## Results

### Analysis of Illumina test dataset

**Fig. 2:**
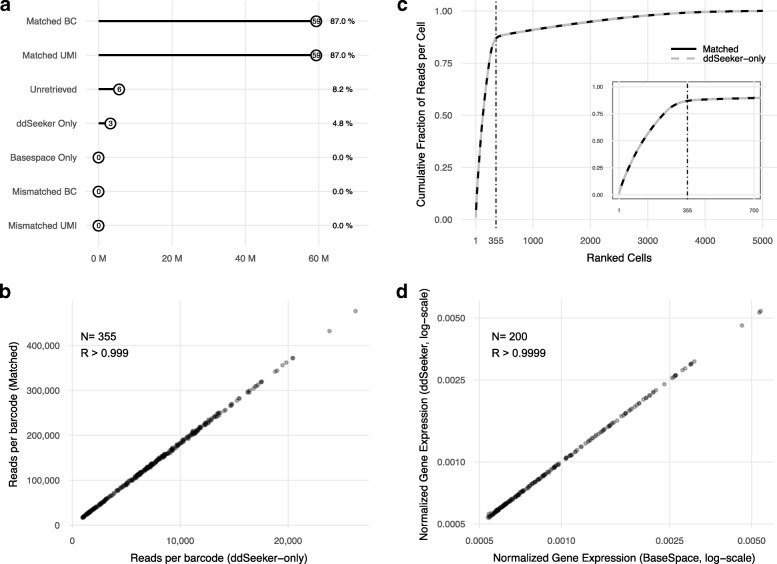
Application of ddSeeker to Illumina test dataset **a** Dot plot reporting the number of reads for which ddSeeker and BaseSpace identify the same barcode and UMI (Matched BC and UMI), ddSeeker and BaseSpace do not identify valid barcode and/or UMI (unretrieved), only ddSeeker or BaseSpace were able to identify barcode and UMI (ddSeeker- and BaseSpace- only) and the number of reads identified with different barcode and/or UMI by the two tools (Mismatched BC and UMI). **b** Cumulative fraction of reads per cell in the 5,000 most read barcodes for matched BC reads (solid black line) and ddSeeker-only reads (dashed grey line). Vertical line corresponds to the number of valid cells based on the knee analysis from the Illumina BaseSpace report (n=355). **c** Scatter plot of the number of reads with matched BC versus the number of ddSeeker-only reads for the 355 valid cells. R is the Pearson’s correlation coefficient. **d** Scatter plot of the averaged normalized expression across the 100 most read cells of the 200 most expressed human genes following ddSeeker (y-axis) versus BaseSpace (x-axis) pipelines

To assess the performance of ddSeeker, we considered a test dataset provided by Illumina obtained from human embryonic kidney 293 (HEK 293) cells and NIH 3T3 mouse embryonic fibroblasts mixed at a 1:1 ratio (N=1400 cells in total). In this study, we considered one of the four replicates (sample A, N=350 cells), that includes 68.2 paired-end million reads. Figure 2 reports the results of the comparative analysis between ddSeeker and BaseSpace. Overall, ddSeeker and BaseSpace identified 62.6 (91.8%) and 59.4 (87.0%) million reads with valid barcodes, respectively (Fig. 2a). The distribution of ddSeeker’s error tags is reported in Additional file 1: Table S2 and shows that the most relevant errors were alignment error in linker 2 (L2, 1.94%), followed by error in barcodes (B, 1.86%) and alignment error in both linkers (LX, 1.54%). To evaluate the performance of ddSeeker in retrieving valid barcodes, we performed a read-by-read comparison of cell barcodes and UMI identified by ddSeeker and BaseSpace. Considering BaseSpace results as a reference, we computed the percentage of reads identified by ddSeeker with the same cell barcode and UMI than BaseSpace. We found that ddSeeker was able to correctly retrieve 100% of barcodes and UMI with no mis-identification. About 8% of reads were flagged as reads with no valid barcode or UMI by both the algorithms. Of note, we found that ddSeeker was able to retrieve 5% more reads with valid barcodes than BaseSpace (Fig. 2a). We verified that all of these reads showed insertions or deletions in the sequence of the linkers or barcodes.

#### Quality assessment of ddSeeker’s additionally detected reads

To evaluate the quality of barcodes exclusively identified by our tool, we first studied the cumulative fraction of reads per cell in the matched (i.e., barcodes identified by both BaseSpace and ddSeeker) and ddSeeker-only barcodes. As reported in Fig. 1b, we found an exact overlap between the two curves, both showing the knee at about 350 cells, as expected based on Illumina BaseSpace report. We also investigated whether considering insertions and/or deletions in our pipeline could introduce biases in the quantification of valid barcodes (i.e., the presence of certain barcodes showing more insertions or deletions than expected). As reported in Fig. 1c, we observed that the number of ddSeeker-only reads linearly correlates with the number of valid barcode reads identified by both algorithms (*R*>0.999). We then studied how the ddSeeker pipeline impacts on downstream analysis, including read alignment and gene counting. First, we evaluated whether the additionally detected reads mapped equally well to the reference genome. To achieve that, we compared mapping quality values extracted from the Illumina bam file for matched and ddSeeker-only valid barcode reads. We found that 72% of ddSeeker-only valid reads has high mapping quality and, in general, the mapping quality distribution for matched and ddSeeker-only valid reads were markedly similar (Additional file 1: Figure S2). Secondly, we investigated the number of doublets detected using ddSeeker and BaseSpace and observed no difference (n=13 for both pipelines, see Additional file 1: Figure S3), demonstrating that additionally detected reads show high species-specificity. Lastly, we compared gene expression estimations following ddSeeker and BaseSpace pipelines. To calculate gene counts, we used the DigitalExpression tool included in Drop-seq tools. A library size normalization (i.e., gene counts per cell) was applied to quantify gene expression levels. Figure 2d reports the mean gene expression level for the 200 most expressed human genes across the 100 most read cells using ddSeeker and BaseSpace (results for mouse genes are reported in Additional file 1: Figure S4). We obtained high correlation (*R*>0.999) between ddSeeker and BaseSpace, demonstrating that overall gene expression estimation is not biased by additionally detected reads by ddSeeker.

### Application of ddSeeker to in-house dataset

We tested ddSeeker further, in the analysis of an in-house dataset that includes 6 scRNA-seq libraries of the MDA-MB-361 breast cancer cell line. Details about cell culture, library preparation and sequencing are reported in the Additional file 1. Our dataset was generated by two Illumina runs characterized by low (cluster saturation) and high sequencing quality (Table 1). FastQC analysis [35] of a subset of R1 (*N*=10*M* reads for both low and high quality read sets) is reported in Additional file 1: Figure S5.

**Table 1:** Sequencing run summary of the in-house dataset

	Run 1	Run 2
Quality	Low	High
# Libraries	6	6
Clusters PF (%)	33.05	93.9
Q30 (%)	70.9	82.6
Total Reads	112993193	197600946
Valid Reads	70826303 (63%)	180526539 (91%)
# Cells (expected)	1800	1800
# Cells (ddSeeker)	≈3000	≈3000

Valid reads are reads with valid barcodes and UMI. Expected number of cells is based on cell capture efficiency, as declared by Bio-Rad

**Fig. 3:**
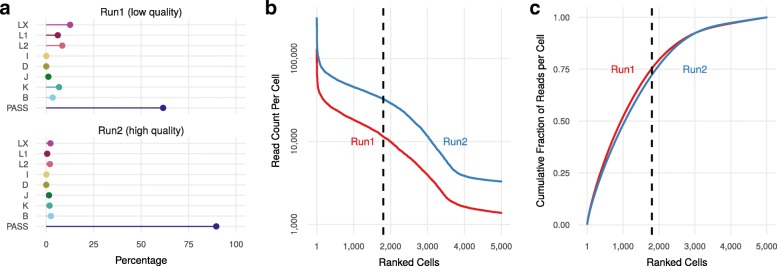
Application of ddSeeker to in-house dataset **a** Dot plot of the percentage of valid reads (PASS) and reads with errors in barcode and/or UMI in the low (Run 1) and high (Run 2) sequencing quality read sets. Details regarding error classification are reported in Additional file 1. **b** Number of reads per cell barcodes across the 5000 most read barcodes in the two read sets. Vertical dashed line indicates the number of expected cells (1800) in our libraries based on ddSEQ specifics. **c** Cumulative fraction of reads per cell in the 5000 most read barcodes in the two read sets. All these plots can be generated with make_graphs.R, a dedicated R script included in the ddSeeker package

Using a 40-core machine, ddSeeker takes approximately 7.0 h to complete the analysis on 310,594,139 million reads from both runs, corresponding to about 1.1 million reads/cpu processed per hour. About 63% and 91% of the total reads were identified as valid in Run 1 (low quality) and Run 2 (high quality), respectively. Figure 3a and Additional file 1: Table S3 report the classification of the errors found by ddSeeker in the R1 of the two runs, and show that error tag distribution in Run 2 was comparable with that one obtained in the Illumina test dataset. We also found that the error tag distributions were the same across the different scRNA-seq libraries (Additional file 1: Figure S6). ddSeeker can output a text file reporting the number of valid reads per cell which can be useful to preliminary estimate the number of sequenced cells before computationally intensive steps such as read alignment, processing and gene counting (Fig. 3b). The number of reads obtained in the 5000 most read barcodes for the two runs is reported in Fig. 3c. Despite the difference between the two read sets in terms of sequence quality, the curves showed a similar trend.

## Conclusions

ddSeeker is, to our knowledge, the first freely-available tool to perform initial processing and quality metrics of reads generated through Bio-Rad ddSEQ/Illumina experiments. We performed a comparative study of ddSeeker and BaseSpace in the analysis of an Illumina test dataset. We showed that ddSeeker was able to identify 5% more reads with valid barcodes and UMI than the Illumina BaseSpace tool. The enhanced ability of ddSeeker in identifying reads with valid barcodes derives from its exclusive feature that implements the analysis of insertions or deletions events in the sequences of linkers and barcodes. Additionally, we demonstrated the reliability of ddSeeker’s additionally detected reads. Our analyses show that downstream analysis is not biased in terms of mapping quality, presence of doublets and gene expression quantification. Finally, we showed the utility of ddSeeker in the analysis of an in-house dataset that includes two different read sets characterized by low and high sequencing quality. To conclude, our analyses suggest that ddSeeker is a valuable tool to perform quality control of Bio-Rad ddSEQ data, and to identify valid reads for downstream scRNA-seq data analysis.

## Availability and requirements

**Project name:** ddSeeker **Project home page:**https://github.com/cgplab/ddSeeker**Operating system:** GNU/Linux **Programming language:** Python 3 **License:** GPL-3.0

## Additional file

Additional file 1 Supplementary text, figures and tables. Supplementary text 1. Classification of error tags. Supplementary text 2. Detailed algorithm description. Supplementary figure 1. Linker alignment analysis. Supplementary figure 2. Error tags distribution for the in-house dataset. Supplementary table 1. Linker alignment analysis. Supplementary table 2. Error tags distribution for the Illumina dataset. Supplementary table 3. Error tags distribution for the in-house dataset. (PDF 407 kb)

## Abbreviations

BC1: Barcode block 1
BC2: Barcode block 2
BC3: Barcode block 3
UMI: Unique molecular identifier
scRNA-seq: Single cell RNA sequencing
uBAM: Unmapped BAM
