# Supplementary material for: ddSeeker: a tool for processing Bio-Rad ddSEQ single cell RNA-seq data
Source: BMC Genomics. 2018 Dec 24;19:960. doi: 10.1186/s12864-018-5249-x (PMC6304778; doi:10.1186/s12864-018-5249-x)
Supplement: Supplementary file 1 — Supplementary text, figures and tables. Supplementary text 1. Classification of error tags. Supplementary text 2. Detailed algorithm description. Supplementary figure 1. Linker alignment analysis. Supplementary figure 2. Error tags distribution for the in-house dataset. Supplementary table 1. Linker alignment analysis. Supplementary table 2. Error tags distribution for the Illumina dataset. Supplementary table 3. Error tags distribution for the in-house dataset. (PDF 407 kb) [file 12864_2018_5249_MOESM1_ESM.pdf]

Supplementary Materials for ”ddSeeker: a tool for processing Bio-Rad ddSEQ single cell RNA-seq data”

Supplementary text

1 Error classification **2**

2 Algorithm description **2**

3 Materials **2**

List of Supplementary Figures

1 Linker alignment analysis 4
2 Mapping quality distribution analysis 5
3 Doublet analysis 6
4 Gene expression analysis 6
5 FastQC analysis of the in-house dataset 7
6 Error tags distribution for the in-house dataset 9

List of Supplementary Tables

1 Linker alignment analysis 7
2 Error tags distribution for the Illumina dataset 8
3 Error tags distribution for the in-house dataset 8

1 Classification of error tags (XE)

LX None of the two linkers can be aligned to the R1 sequence with less than one mismatch (including indels).

L1 Linker 1 cannot be aligned to the R1 sequence with less than one mismatch (including indels).

L2 Linker 2 cannot be aligned to the R1 sequence with less than one mismatch (including indels).

I The distance between the two linkers is below 5 or above 7 (Indels in BC2).

D The starting position of the linker 1 is less then 5 nucleotides from the start of the R1 (Deletion in Phase Block or BC1).

J The trinucleotide upstream of UMI has a sequence that differs from ACG for more than one mismatch (Indels upstream/within the trinucleotide or mismatches affecting the trinucleotide).

K The trinucleotide downstream of UMI has a sequence that differs from GAC for more than one mismatch (Indels upstream/within the trinucleotide or mismatches affecting the trinucleotide).

B At least one of the three barcode blocks cannot be aligned to any of the known barcodes with less than one mismatch (including indels).

2 Algorithm description

Position of linkers The two linkers are aligned to the sequence of R1 using the align.localxs function (gap parameters: -2, -1) from Biopython/pairwise2 module. If at most one mismatch (or gap) is found, the starting positions are retrieved. The sequences of the remaining elements of the R1 are then retrieved with respect to the starting positions of either one of the two linkers. A correction factor k (deletion: -1; insertion: +1; no indels: 0) is stored for each linker to correct the relative positions of such elements.

Distance between linkers The difference between the starting points of the two linkers, plus the k factor, is verified to be within 20-22 (linker length + BC2 length + k = 15 + 6 + -1/0/+1).

Linker 1 starting position Linker 1 is verified to start at least 5 nucleotides from the start of R1 (length of BC1 + one deletion).

Trinucleotides The sequence of the two trinucleotides upstream and downstream of the UMI and verified to be ACG and GAC respectively. In each case, at most one mismatch but no gaps are allowed.

Barcode blocks comparison The three barcode blocks (BC1, BC2, BC3) are aligned to a set of known barcode blocks using the align.globalxs function (gap parameters: -1, -1) from Biopython/pairwise2 module. If at most one mismatch (or gap) is found, the sequence of the known block is then assigned to the corresponding BC.

Return results The UMI sequence and the combination of the three BCs are assigned to the XU and XB tags, respectively.

3 Cell Culture, Library preparation and sequencing

MDA-MB-361 breast cancer cell line was purchased from Sigma-Aldrich. Cells were cultured in Dulbecco's modified Eagles medium (DMEM) with 4.5g/glucose and L-glutamine (Lonza) supplemented with 10% heat-inactivated fetal bovine serum (FBS) (Hyclone) and 10,000 U penicillin and 10 mg steptomycin/mL solution (P/S) (Sigma-Aldrich). MDA-MB-361 cells were found to be mycoplasma-negative using MycoAlert Mycoplasma Detection kit (Lonza) and authenticated with the short tandem repeat analysis performed in service by BMR Genomics, Padova, Italy. Cells were encapsulated into nanoliter droplets and single cell transcriptomes barcoded using Bio-Rad ddSEQ Single Cell Isolator

(Bio-Rad). Briefly, to obtain a single cell suspension, a 60-90% confluent 25 cm^2 plate of MDA-MB-361 was trypsinized, filtered through a 35μm nylon strainer (Falcon) and diluted to 2500 cells/μl in 1XPBS (Gibco) + 0.1 BSA (Santa Cruz Biotechnology) solution. We only proceeded with samples showing $> 95\%$ viability, according to the manufacturer's recommendations. Cell count and viability were evaluated with phase contrast microscope (Leica) and automated cell counter (Invitrogen). Finally, 4.5 μl of the cell suspension were loaded in each well of one ddSEQ M cartridge (Bio-Rad) in order to obtain an average of 300 single cells per chamber. cDNA library preparation was performed following the Illumina Bio-Rad SureCell WTA 3' Library kit (Illumina). Quantification and quality analysis of cDNA libraries were carried out on Agilent 2100 Bioanalyzer system (Agilent) using High Sensitivity DNA chips (Agilent). Libraries were sequenced on a Hiseq2500 Rapid Run Mode at Centre for Integrative Biology (CIBIO) Next Generation Sequencing (NGS) Facility, Trento, Italy, following protocol recommendations (Illumina); The bases per reads were: R1 (68) - I (8) - R2 (75); 10% PhiX per lane was included and R1 Custom Seq Primer was spiked in HP10.

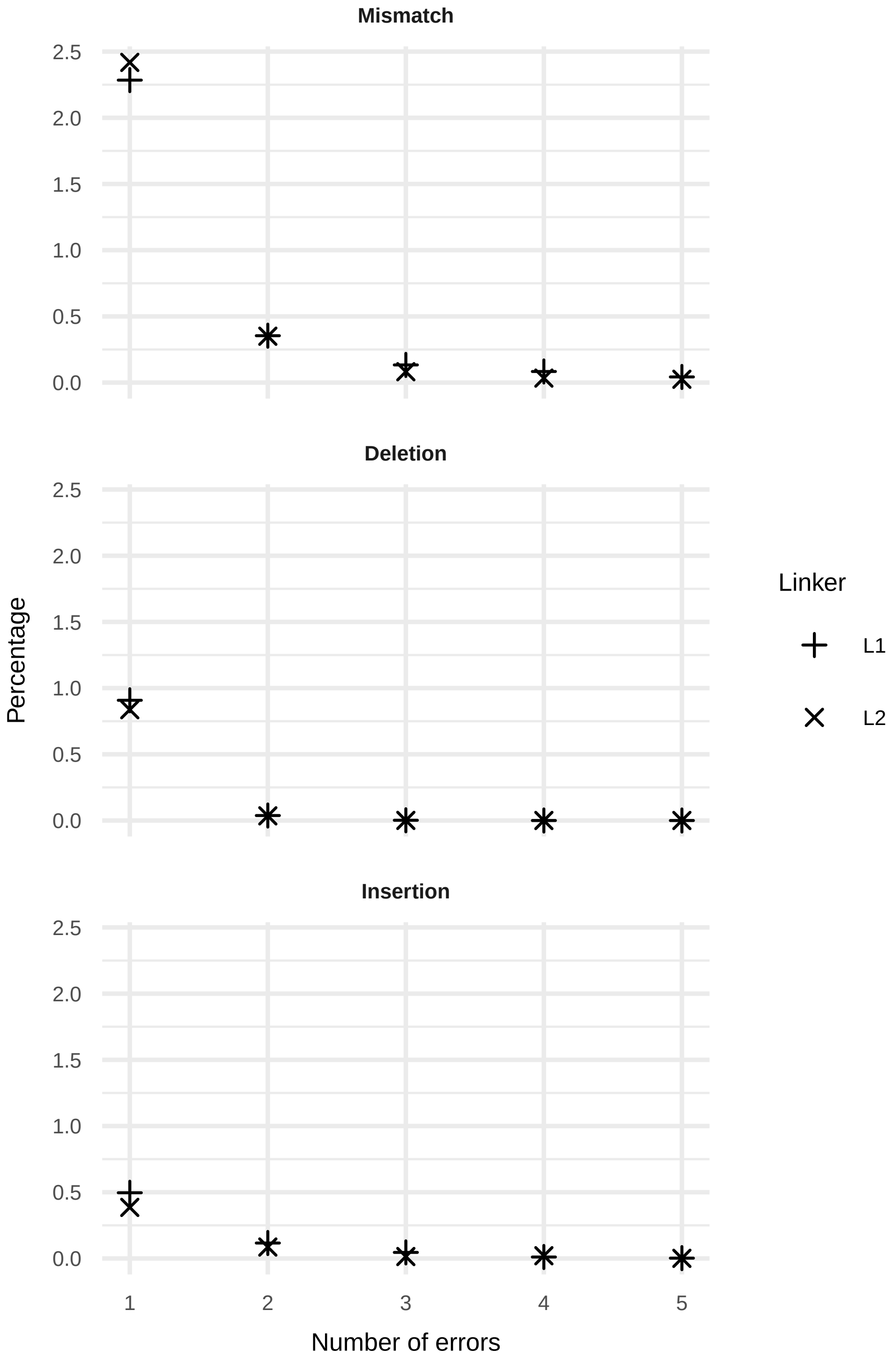

Figure 1: Distribution of the percentage of reads having alignment errors (mismatches, insertions and deletions) in their linker 1 and 2. Data are from the Illumina test dataset. Data related to more than 5 alignment errors are reported in Supplementary Table 3.

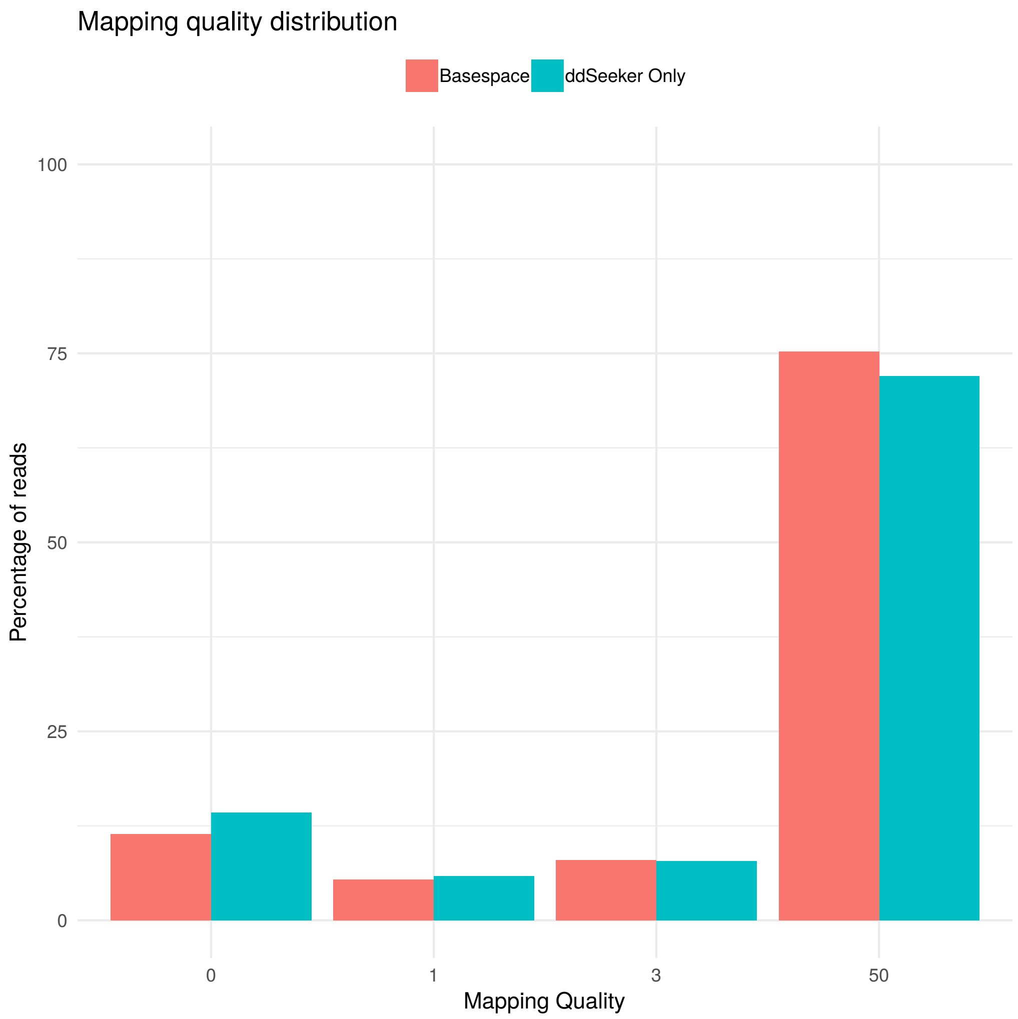

Figure 2: Bar plots showing the percentage of reads with different mapping quality values for BaseSpace (red bars) and ddSeeker-only reads (light blue bars). Mapping quality values were extracted from the Illumina bam file.

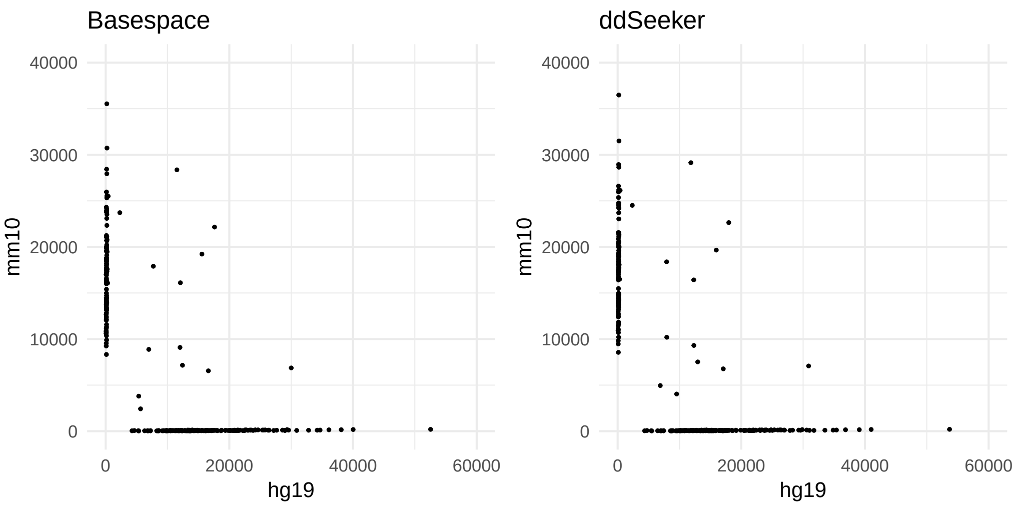

Figure 3: Scatter plot of the number of UMI per cell aligned to human (hg19, x-axis) and mouse (mm10, y-axis) genes after BaseSpace (left) and ddSeeker (right) pipeline. Doublets (n=13 for both analyses) were estimated as those cells having more than 4000 UMI in both human and mouse genes. The number of UMI per cell was evaluated with the DigitalExpression tool included in Drop-seq tools.

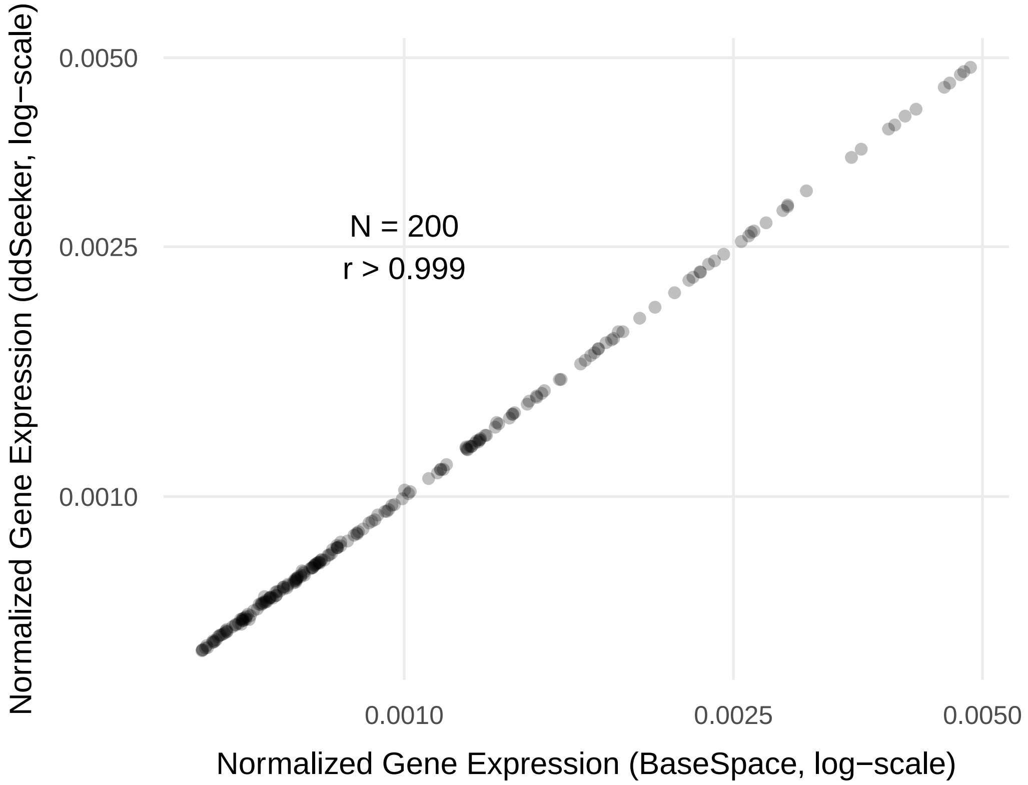

Figure 4: Scatter plot of the averaged normalized expression across the 100 most read cells of the 200 most expressed mouse genes following ddSeeker (y-axis) versus BaseSpace (x-axis) pipelines.

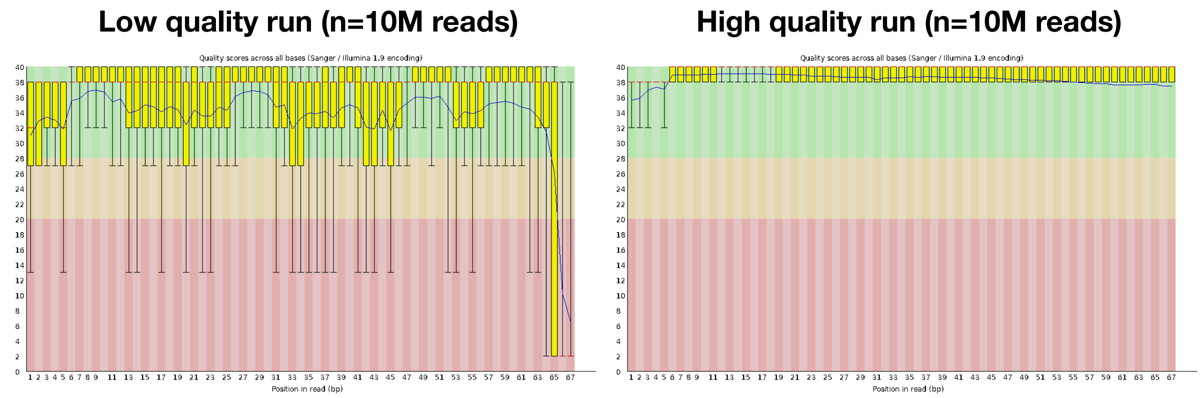

Figure 5: FastQC analysis of 10 million reads of the low (left) and high (right) sequencing quality read sets. Data are from the reads 1 of the in-house dataset.

Table 1: Summary statistics for alignment errors in linker 1 and linker 2. Data are from the Illumina test dataset.

Linker	Type	Errors	Count	%
L1	Mismatch	1	1558869	2.28
L1	Mismatch	2	241613	0.35
L1	Mismatch	3	90936	0.13
L1	Mismatch	4	57224	0.08
L1	Mismatch	≥ 5	416430	0.61
L1	Deletion	1	619613	0.91
L1	Deletion	2	25714	0.04
L1	Deletion	3	1145	0.00
L1	Deletion	4	2	0.00
L1	Deletion	≥ 5	0	0.00
L1	Insertion	1	338421	0.50
L1	Insertion	2	79427	0.12
L1	Insertion	3	31045	0.05
L1	Insertion	4	7296	0.01
L1	Insertion	≥ 5	1505	0.00
L2	Mismatch	1	1650200	2.42
L2	Mismatch	2	238819	0.35
L2	Mismatch	3	55947	0.08
L2	Mismatch	4	23037	0.03
L2	Mismatch	≥ 5	357603	0.52
L2	Deletion	1	571094	0.84
L2	Deletion	2	23930	0.04
L2	Deletion	3	452	0.00
L2	Deletion	4	0	0.00
L2	Deletion	≥ 5	0	0.00
L2	Insertion	1	262959	0.39
L2	Insertion	2	58670	0.09
L2	Insertion	3	9822	0.01
L2	Insertion	4	13910	0.02
L2	Insertion	≥ 5	450	0.00

Table 2: Distribution of tag events (errors and passing R1) in the Illumina test dataset.

Type	# Events	%
Illumina		
LX	1048661	1.54
L1	354226	0.52
L2	1324283	1.94
I	35766	0.05
D	6815	0.01
J	603353	0.88
K	974900	1.43
B	1271235	1.86
PASS	62614642	91.77

Table 3: Distribution of tag events (errors and passing R1) in the low and high quality runs.

Run 1			Run 2		
Type	# Events	%	Type	# Events	%
LX	14204901	12.57	LX	4403022	2.23
L1	6871148	6.08	L1	985026	0.50
L2	9541248	8.44	L2	3838653	1.94
I	66960	0.06	I	186707	0.09
D	7423	0.01	D	19085	0.01
J	1259649	1.11	J	2903907	1.47
K	7633089	6.76	K	3481732	1.76
B	3842453	3.40	B	4811643	2.44
PASS	69566322	61.57	PASS	176971171	89.56

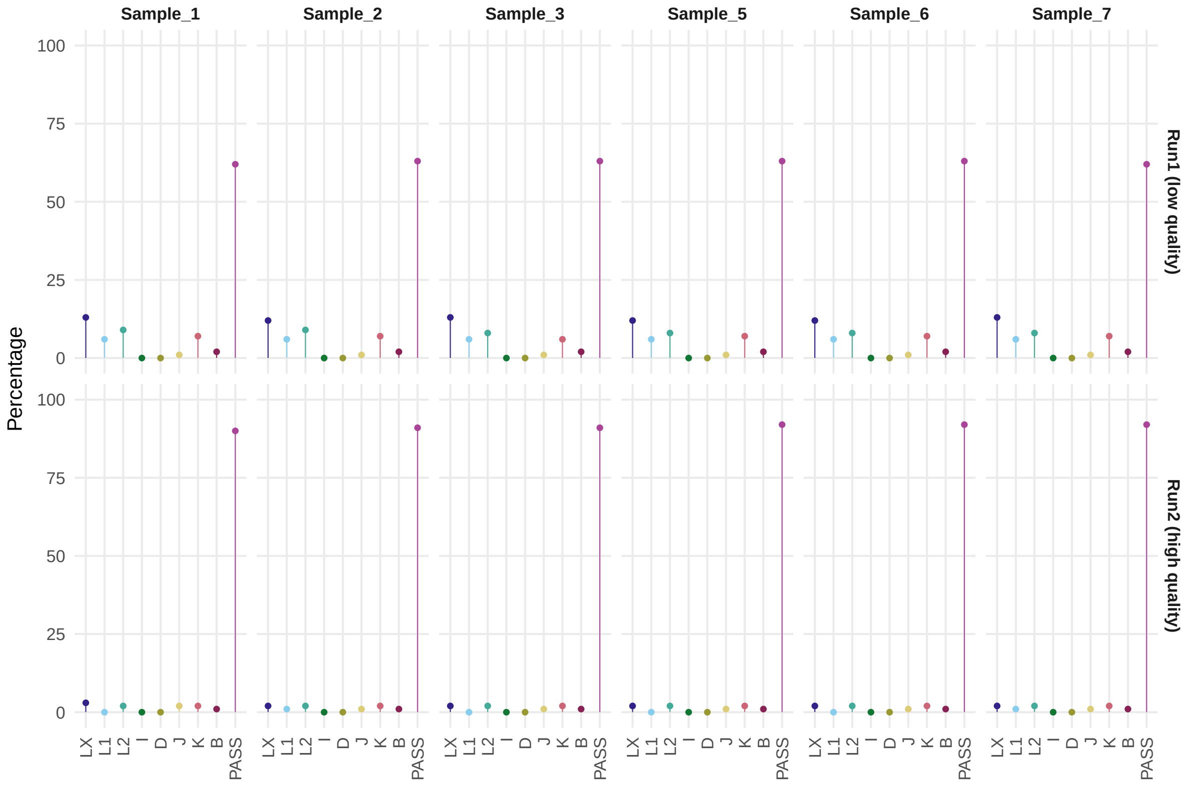

Figure 6: Distribution of alignment errors across the different scRNA-seq libraries (1,2,3,5,6,7) in the low quality run (Run 1, top row) and in the high quality run (Run 2, bottom row).
